# Raw Milk Microbiota Modifications as Affected by Chlorine Usage for Cleaning Procedures: The Trentingrana PDO Case

**DOI:** 10.3389/fmicb.2020.564749

**Published:** 2020-10-06

**Authors:** Paola Cremonesi, Stefano Morandi, Camilla Ceccarani, Giovanna Battelli, Bianca Castiglioni, Nicola Cologna, Andrea Goss, Marco Severgnini, Massimiliano Mazzucchi, Erika Partel, Alberto Tamburini, Lucio Zanini, Milena Brasca

**Affiliations:** ^1^Institute of Agricultural Biology and Biotechnology, Italian National Research Council, Lodi, Italy; ^2^Institute of Sciences of Food Production, Italian National Research Council, Milan, Italy; ^3^Institute of Biomedical Technologies, Italian National Research Council, Segrate, Italy; ^4^Department of Health Sciences, San Paolo Hospital Medical School, University of Milan, Milan, Italy; ^5^Trentingrana–Consorzio dei Caseifici Sociali Trentini s.c.a., Trento, Italy; ^6^Technology Transfer Center, Edmund Mach Foundation, Trento, Italy; ^7^Department of Agricultural and Environmental Sciences, Faculty of Agricultural and Food Sciences, University of Milan, Milan, Italy; ^8^Breeders Association of Lombardy, Crema, Italy

**Keywords:** chlorine, whey-starter, cheese, milking equipment, biodiversity

## Abstract

Milk microbiota represents a key point in raw milk cheese production and contributes to the development of typical flavor and texture for each type of cheese. The aim of the present study was to evaluate the influence of chlorine products usage for cleaning and sanitizing the milking equipment on (i) raw milk microbiota; (ii) the deriving whey-starter microbiota; and (iii) Trentingrana Protected Designation of Origin (PDO) cheese microbiota and volatilome. Milk samples from three farms affiliated to a Trentingrana PDO cheese factory were collected three times per week during a 6-weeks period in which a sodium hypochlorite detergent (period C) was used and during a subsequent 6-weeks period of non-chlorine detergent usage (period NC). Samples were subjected to microbiological [Standard Plate Count; coliforms; coagulase-positive staphylococci; and lactic acid bacteria (LAB)] and metagenomic analysis (amplification of V3-V4 regions of 16S rRNA gene performed on Illumina MiSeq platform). In addition, cheese volatilome was determined by SPME-GC-MS. In the transition from period C to period NC, higher SPC and LAB counts in milk were recorded. Milk metagenomic analysis showed a peculiar distinctive microbiota composition for the three farms during the whole experimental period. Moreover, differences were highlighted comparing C and NC periods in each farm. A difference in microbial population related to chlorine usage in bulk milk and vat samples was evidenced. Moreover, chlorine utilization at farm level was found to affect the whey-starter population: the usually predominant *Lactobacillus helveticus* was significantly reduced during NC period, whereas *Lactobacillus delbrueckii* had the exact opposite trend. Alpha- and beta-diversity revealed a separation between the two treatment periods with a higher presence of *L. helveticus*, *L. delbrueckii*, and *Streptococcus thermophilus* in cheese samples after NC detergent period. Cheese volatilome analysis showed a slight decrease in lipolysis during C period in the inner part of the cheese wheel. Although preliminary, these results suggest a profound influence on milk and cheese microbiota, as well as on raw milk cheese production and quality, due to the use of chlorine. However, further studies will be needed to better understand the complex relationship between chlorine and microbiota along all the cheese production steps.

## Interpretative Summary

Chlorine compounds are commonly used in farms for sanitation purposes due to their bactericidal activity, but attention has been be paid to the effect of formation of chlorinated residues with an impact on milk. This study is aimed at evaluating the influence of chlorine products on raw milk microbiota and cheese flavor through metagenomic analysis and cheese volatilome investigation in farms associated to Trentingrana PDO cheese production. Our preliminary results support the idea that the detergents used influence raw milk microbial population and, consequently, raw milk cheese quality, safety, and sensory attributes. However, further studies will be needed to better understand the complex relationship between detergent and microbiota along all the steps of cheese production.

## Introduction

Chlorine compounds are commonly used in farms, water treatment, and industrial food manufacturing processes, for sanitation purposes (McCarthy et al., 2018). These compounds, such as sodium hypochlorite (NaClO), calcium hypochlorite (Ca(ClO)_2_) and chlorine dioxide (ClO_2_), possess a high bactericidal activity against food-borne pathogens and, under proper conditions, may prevent the formation of biofilm in milking installation (Gómez-López et al., 2009; Sundberg et al., 2011).

The antimicrobial mechanism of chlorine compounds has been studied by several investigators. The most widely accepted mechanism is a damage to the protein synthesis and an increased permeability of the outer cell membrane (Gómez-López et al., 2009). Previous studies demonstrated that NaClO is effective for the milking equipment disinfection, reducing the bacteria populations by 99% on stainless steel surfaces (Greene et al., 1993; Reinemann et al., 2003). In addition, the incorporation of NaClO into cleaning products guarantees the removal of protein deposits and improves the cleaning efficiency on milking plant surfaces (Reinemann et al., 2003). For this reason, a high portion of liquid products used for milking machines cleaning and sanitation contain sodium hypochlorite (Gleeson et al., 2013). However, it has to be considered that the use of chlorine-based products, although effective and inexpensive, leads to the formation of chlorinated residues [i.e., Trichloromethane (TCM) and Chlorate], with associated health concerns that can rise to dangerous levels in milk fat products such as cream and butter, and, thus, are the subject of attention by food regulatory authorities (EFSA Panel on Contaminants in the Food Chain (CONTAM) Gleeson et al., 2013; Ryan et al., 2013; EFSA Panel on Contaminants in the Food Chain (CONTAM), 2015; McCarthy et al., 2018). In addition, the presence of chlorine can potentially influence the composition of the microbiota in milk and milk products.

Raw milk harbors one of the most diverse microbial communities detected in food matrixes (Quigley et al., 2013). The bacterial species present in raw milk depend on numerous biotic and abiotic drivers that influence and assembly the milk microbiota. Recent advances provided that, along with environment, the teat skin is one of the major source of microbial contamination of raw milk. Bacteria belonging to the families *Clostridiaceae*, *Staphylococcaceae*, *Lactobacillaceae*, *Streptococcaceae*, *Enterococcaceae*, and *Pseudomonadaceae* may reach the milk from the teat surface (Doyle et al., 2017; Frétin et al., 2018). Other factors crucial for microbial contamination are the stage of lactation and seasonality and the dairy environment (feed, feces, humans, and air) (Zucali et al., 2011; Gobbetti et al., 2018). Moreover, the biofilms that may grow in milking equipment could be a vehicle of bacterial transfer in the dairy chain (Marchand et al., 2012). It is difficult to establish the relative importance of these sources of contamination, but each of them can influence the raw milk microbiota and, consequently, the microbial composition of bulk milk, whey starter and raw milk cheese.

In the light of the above evidence, it is important to find alternatives to the use of chlorine in dairy farms and to evaluate the potential effects of chlorine substitution, even temporary, on the milk microbiota and the presence of undesirable microorganisms. Moreover, it is of interest to deepen the relationships between the use of chlorine and the sensory characteristics of raw milk cheeses.

Trentingrana is a Protected Designation of Origin (PDO) cheese, produced in a specific alpine area of Northern Italy and its label belongs to Grana Padano PDO cheese consortium (EU, 2009). It is a hard-textured, cooked, and long-ripened (9–30 months) cheese made using raw cow milk supplemented with natural whey culture (NWC). NWC is obtained from the spontaneous fermentation (44–45°C) of the whey drained from the cheese vat at the end of cheese-making. The microbiota which develops from curd to Trentingrana ripened cheese arises only from raw milk and from NWC (Rossi et al., 2012).

The aim of this study was to determine the effect of chlorine (NaClO) use in the cleaning of milking machine on the microbiota of raw milk, NWC and cheese in Trentingrana production, in normal conditions of use.

## Materials and Methods

### Farms Characteristic and Experimental Plan

Three farms, indicated as F1, F2, and F3, respectively, located in Trentino (northeastern Italian Alps, at an altitude of about 1000 m above sea level) at a distance less than 10 km and associated to a factory producing Trentingrana PDO cheese, were involved in this study.

The experimentation was conducted over a 3 months period (from 5th December 2016 to 12th March 2017) during which the temperature variation during the day in the area where the farms and the dairy plant are located remained stable (between −2 and 9°C on the first day and between −4 and 8°C on the last day).

Two weeks before the start of the experiment each farm was preliminary visited to verify milking equipment, milking routine, washing and cooling system. In order to verify the effectiveness of cleaning procedures (with chlorine) of milking equipment and to establish a common procedure, the Lactocorder analysis was used (Bava et al., 2011).

The herds of each farm were composed by 55, 48, and 91 lactating cows (Italian Holstein-Friesian, Brown and Italian Simmental in different ratios), housed in free stall barns with cubicles, with mattress covered with sawdust; lactating cows were fed hay produced in the production area of Trentingrana and associated with concentrate. The herds were milked twice a day in DeLaval herringbone milking plants [F1 (5 + 5), F2 (4 + 4), F3 (6 + 6)]. Dairy farmers used gloves during milking. A commercial product including only detergent and emollient agents, was used for the pre-milking teat dip, and the teats were dried with disposable paper towels before the forestripping and the attachment of the milking cups. At the end of the milking a post-dipping product including lactic acid was applied. The milking equipment had automatic wash facilities ensuring uniformity and consistency of sanitizing practices.

Each auto-washer was calibrated for the cleaning product before the start date. The cleaning routine practices and products usage rates were those recommended by the producer and the water temperatures appropriate (>60°C). In the first experimental period (first 5-weeks period, named C) sodium hypochlorite (NaClO) detergent was used and in a subsequent, analogous, period (last 5-weeks period, named NC) non-chlorine liquid alkaline detergent (Perlac, Perdomini, Italy) was utilized. A 4-weeks interval was established between C and NC experimental periods in order to allow bacterial population adaptation to the new detergent.

Bulk tank milk samples were collected from three dairy farms over a 3 months period (from December 2016 to March 2017). Samples were collected in the last 3 days of each experimental week during C and NC period. One hundred-milliliter were collected from the storage tanks of each farm in sterile vials without preservative. In each sampling day, the milk obtained from the three farms was transported to a dairy factory, pooled, and, after natural creaming, used for the production of Trentingrana cheese. The whole bulk milk from the evening milking (WM), the corresponding partially skimmed milk obtained after overnight natural creaming (SM) and natural whey starter samples used for cheesemaking were collected for analysis.

Milk and whey samples intended for microbiological analysis were cooled at 4°C and analyzed within 8 h, while the samples for microbiota analysis were frozen and, then, transferred to the laboratory. Three cheese wheels for each experimental period were subjected to metagenomic and volatilomic analysis after 12 months of ripening.

A total of 45 milk samples (15 for each farm) were analyzed both for C and NC period along with 15 WM and 15 SM and 15 natural whey starters.

### Milk and Natural Whey Culture Samples Microbiological Analysis

Milk samples were serially diluted in quarter-strength Ringer’s solution (Scharlau Microbiology, Barcelona, Spain) and analyzed for standard plate count, coliforms and coagulase-positive staphylococci according to the procedures of the International Organization for Standardization (ISO, 1999, 2006, 2013). Lactic acid bacteria (LAB) were counted on de Man Rogosa and Sharpe (MRS) agar (Biolife Italiana, Milano, Italy) after anaerobic incubation (Anaerocult A, Merck, Darmstadt, Germany) at 30°C for 72 h and in M17 Agar (Biolife Italiana) incubated at 30°C in aerobic condition for 48 h. Heterofermentative LAB were determined by the most probable number (MPN) method using MRS broth with Durham tubes (MRS + C). Inoculated tubes were incubated at 30°C for 72 h and later at 37°C for 48 h and examined daily for gas production (Morandi et al., 2019). Positive tubes were checked microscopically to exclude the presence of yeasts and Enterobacteriaceae.

The MPN results were evaluated according to ISO 7218 (ISO, 2013).

Whey cultures were serially diluted in reconstituted (10% w/v) skimmed milk (Sacco System, Cadorago, Italy) and inoculated in Plate Count Agar (Biolife Italiana) under anaerobic conditions (Anaerocult A) at 44°C for 72 h.

The analyses were carried out in triplicate, and microbiological data were transformed by logarithm base 10 (log) and expressed in the descriptive statistic as mean and standard deviation (Std). A GLM analysis was performed with experimental period and month of analyses as fixed effects.

### DNA Extraction and Purification

Milk and whey samples were thawed at room temperature. Five ml of milk were centrifuged at 500 × *g* for 10 min at 4°C; the supernatant was discarded, and the pellet was resuspended with 1 ml of saline solution (NaCl 0.9%) and centrifuged at 500 × *g* for 5 min at 4°C. The supernatant was discarded and the bacterial DNA was extracted from the samples as described previously (Cremonesi et al., 2006, 2018), by using a method based on the combination of a chaotropic agent (i.e., guanidinium thiocyanate) with silica particles, to obtain bacterial cell lysis and nuclease inactivation. For Trentingrana PDO cheese (three samples from each treatment), 45 mL of 2% (w/v) K_2_HPO_4_ buffer solution (Sigma-Aldrich, Milan, Italy) were added to 5 g of cheese; the sample was, then, mixed for 90 s in a Stomacher machine (PBI, Milan, Italy). The DNA was extracted starting from 800 μL of the homogenized sample following the protocol described in Cremonesi et al. (2006) with some modifications. Briefly, 400 μL of lysis buffer (3 mol/L guanidine thiocyanate, 20 mmol/L EDTA, 10 mmol/L Tris–HCl, pH 6.8, 40 mg/mL Triton X-100, 10 mg/mL dithiothreitol) and 300 μL of binding solution (40 mg/mL silica from Sigma Aldrich, directly suspended in the lysis buffer) were added to the sample and vortexed for 30 s to obtain an emulsified solution. Then, the sample was incubated for 5 min at room temperature. After this step, the protocol was the same described in Cremonesi et al. (2006) with centrifugations at 550 × *g*. DNA quality and quantity were assessed using a NanoDrop ND-1000 spectrophotometer (NanoDrop Technologies, Wilmington, DE, United States). The isolated DNA was stored at −20°C until use.

### 16S rRNA Gene Library Construction and Sequencing

Bacterial DNA was amplified using the primers described in literature (Caporaso et al., 2011) which target the V3-V4 hypervariable regions of the 16S rRNA gene. All PCR amplifications were performed in 25 μl volumes per sample. A total of 12.5 μl of Phusion High-Fidelity Master Mix 2× (Thermo Fisher Scientific, Waltham, MA, United States) and 0.2 μl of each primer (100 μM) were added to 2 μl of genomic DNA (5 ng/μl). Blank controls (i.e., no DNA template added to the reaction) were also performed. A first amplification step was performed in an Applied Biosystems 2700 thermal cycler (Thermo Fisher Scientific, Monza, Italy). Samples were denatured at 98°C for 30 s, followed by 25 cycles with a denaturing step at 98°C for 30 s, annealing at 56°C for 1 min and extension at 72°C for 1 min, plus a final extension at 72°C for 7 min. Amplicons were cleaned with Agencourt AMPure XP (Beckman Coulter, Brea, CA, United States) and libraries were prepared following the 16S Metagenomic Sequencing Library Preparation Protocol (Illumina, San Diego, CA, United States). The libraries obtained were quantified by Real Time PCR with KAPA Library Quantification Kits (Kapa Biosystems, Inc., Wilmington, MA, United States), pooled in equimolar proportion and sequenced in one MiSeq (Illumina, San Diego, CA, United States) run with 2 × 250-base paired-end reads.

### Microbiota Profiling

The reads obtained were analyzed by merging pairs using Pandaseq (Masella et al., 2012) and by discarding low quality reads (i.e., >25% bases with a phred Q-score <3). For computational reasons, a subset of 50,000 random reads per sample was extracted. Filtered reads were processed using the QIIME pipeline (v 1.8.0) (Kuczynski et al., 2011), clustered into Operational Taxonomic Units (OTUs) at 97% identity level and taxonomically assigned via RDP classifier (Wang et al., 2007) against the Greengenes database (release 13_8)^1^. Singletons (i.e., OTUs supported by only 1 read across all samples) were discarded as likely chimeras. OTU table was rarefied to the least-sequenced sample (i.e., 30,087 sequences). Alpha-diversity evaluations were performed using Chao1, Shannon index and observed species metrics and rarefaction curves were employed to determine whether most of the bacterial diversity had been captured. Statistical evaluation of differences in microbial alpha-diversity was performed by a non-parametric Monte Carlo-based test using 9,999 random permutations.

For beta-diversity, principal coordinates analysis (PCoA) was performed using weighted and unweighted UniFrac distances. Adonis function, which performs a partitioning of distance matrices among sources of variation using a permutation test with pseudo-F ratios, of the R package vegan (Oksanen et al., 2013) was employed to determine statistical separation of the microbiota profiles. Where appropriate, an analysis of intra- vs. inter- sample distances (i.e., comparison of distances among samples from the same experimental class vs. distances from those of the other class) were performed.

Differences in abundances of bacterial taxa among experimental groups were analyzed by non-parametric Mann–Whitney *U*-test. Unless otherwise reported, a *p*-value of 0.05 was used to assess significance. Statistical elaborations were performed using MATLAB software (R2008b, Natick, MA, United States).

### *Lactobacillus* and *Streptococcus* Species Analysis

Characterization of *Lactobacillus* and *Streptococcus* spp. was performed by re-aligning all reads classified by QIIME within these genera to a custom reference database, which included a total of 149 *Lactobacillus* and 61 *Streptococcus* species with a genome finishing grade of “Complete,” “Chromosome,” or “Scaffolds” in NIH-NCBI database^2^, for a total of 518 and 6,610 strains, respectively. *Lactobacillus* and *Streptococcus* spp. reads were clustered at 100% and re-classified through nucleotide BLAST (legacy BLAST, v 2.26, Altschul et al., 1990), using a *e*-value cutoff of 1e-10 and de-activating the dust-filter. Only reads matching for at least of 80% of their length were retained and, for each read, the best match (i.e., that or those with the higher bit-score) was selected. If a read had multiple classifications on different species, the classification was reset to genus level. Species-level characterization of *Streptococcus* spp. was performed on cheese samples only, whereas, for *Lactobacillus* spp., both whey starter and cheese samples were considered.

### Volatilome Analysis

Cheese samples for the volatilome analysis were obtained from a half slice of the wheel 2.5 cm thick in two different position, 5 cm undercrust (“peripheral”) and 5 cm from the core (“inner”), by means of a cylindrical tester of 10 mm diameter, and immediately introduced in the HS-vial for the analysis.

Volatilome produced by enzymatic activity during cheese ripening was determined by Solid Phase Micro Extraction-Gas Chromatography-Mass Spectrometry (SPME-GC-MS) on the two portions (inner and peripheral) of the cheese. The extraction, separation, identification and semi-quantitation of the volatile compounds was conducted by means of a Combi-Pal automated sampler (CTC Analytics AG, Zwingen, Switzerland) coupled to an Agilent 6890 gas chromatograph with an Agilent 5975 mass spectrometric detector (Agilent Technologies, Santa Clara, CA, United States) and a polar column (Zebron ZB-WAX plus, 60 m × 0.25 mm × 10.25 μm, Phenomenex, Torrance, CA, United States). Extraction and separation conditions were described elsewhere (Battelli et al., 2019). Data were expressed as arbitrary units, as log_10_ of the peak area of the corresponding selected ion.

Statistical analysis was performed with the software package MINITAB ver. 15.1.20 (Minitab Inc., State College, PA, United States). Data were analyzed by ANOVA using the Tukey multiple comparisons method. A *p*-value ≤ 0.05 was considered significant.

### Data Availability

Raw reads for both milk and cheese samples are available in NCBI Short Read Archive (SRA)^3^ under accession number PRJNA616456.

## Results

### Microbiological Quality of Milk and Whey Samples

The first evidence was that not using chlorine in cleaning milking equipment did not lead to any significant increase in coliforms and staphylococci content in milk within the 3-month trial period (Table 1). Bacterial counts tended to be lower in NC period (1.97 ± 1.02 vs. 1.60 ± 0.68 log CFU/mL for coliforms and 2.17 ± 0.48 vs. 2.07 ± 0.57 log CFU/mL for *Staphylococcus aureus*). Moreover, even if no significant differences were observed, higher levels of SPC and LAB in bulk milk were recorded comparing period C to period NC (4.06 ± 0.16 vs. 4.10 ± 0.20 log CFU/mL and). An increase in total bacteria count, although not significant, was observed also in whey-starter (Table 2 – 8.11 ± 0.43 log CFU/mL vs. 8.66 ± 0.45 log CFU/mL in period C and NC, respectively). A slight increase in heterofermentative content was observed moving from C to NC period.

**TABLE 1:** Microbiological quality of bulk milk samples (*n* = 18 for each farm and experimental period).

**Log CFU/mL**	**FARM 1**				**FARM 2**				**FARM 3**				
	**Experimental period**				**Experimental period**				**Experimental period**				
	**C**		**NC**		**C**		**NC**		**C**		**NC**		***p***
	**Mean**	**Std**	**Mean**	**Std**	**Mean**	**Std**	**Mean**	**Std**	**Mean**	**Std**	**Mean**	**Std**	
SPC	4.03	0.10	4.00	0.00	4.11	0.24	4.03	0.13	4.05	0.07	4.25	0.26	NS
Coliforms	1.90	0.36	1.30	0.34	2.35	1.63	1.42	0.69	1.66	0.55	2.09	0.69	NS
*S. aureus*	2.54	0.32	2.33	0.49	1.65	0.23	1.54	0.42	2.31	0.32	2.33	0.38	NS
LAB in MRS	2.33	0.17	2.85	0.39	2.95	1.11	2.69	0.42	2.65	0.34	3.02	0.47	NS
LAB in M17	3.42	0.56	3.30	0.25	3.45	0.99	3.38	0.55	3.95	0.37	4.20	0.76	NS
Heterofermentative LAB	1.43	0.79	1.82	1.08	1.65	0.56	2.05	0.56	1.55	0.88	1.61	0.91	NS

*Data are reported as mean and standard deviation (Std); NS stands for not significant (*P* > 0.05).*

**TABLE 2:** Partially skimmed milk by natural creaming and natural whey starter microbial counts in chlorine (C) and non-chlorine (NC) experimental periods (data are expressed as Log10 CFU/mL).

	**Experimental period**				
	**C**		**NC**		***p***
	**Mean**	**Std**	**Mean**	**Std**	
**Naturally creamed milk**					
SPC	1.00	0.00	1.07	0.13	NS
Coliforms	1.52	0.50	1.50	0.64	NS
LAB in MRS	2.60	0.48	2.57	0.55	NS
LAB in M17	3.13	0.45	3.45	0.73	NS
Heterofermentative LAB	2.02	0.86	1.90	1.06	NS
**Whey starter**					
Anaerobic bacteria	8.11	0.43	8.66	0.45	NS
Heterofermentative LAB	1.82	0.4	2.08	0.86	NS

*Data are reported as mean and standard deviation (Std); NS stands for not significant (*p* > 0.05).*

### Milk and Whey Microbiota Revealed Differences Between Sodium Hypochlorite and Non-chlorine Detergent Period

The microbiota structure of milk and whey-starter samples (*n* = 96) was characterized by a total of 4,789,548 high quality reads, with a mean of 49,891 ± 1,067 reads per sample. Microbial profiles were evaluated: (i) for the three different farms (i.e., F1, F2, F3) and; comparing the experimental period (C, NC) (ii) per farm; (iii) on bulk (whole milk on arrival at the dairy) and vat (partially skimmed milk) milk; (iv) on whey-starter.

**FIGURE 1:**
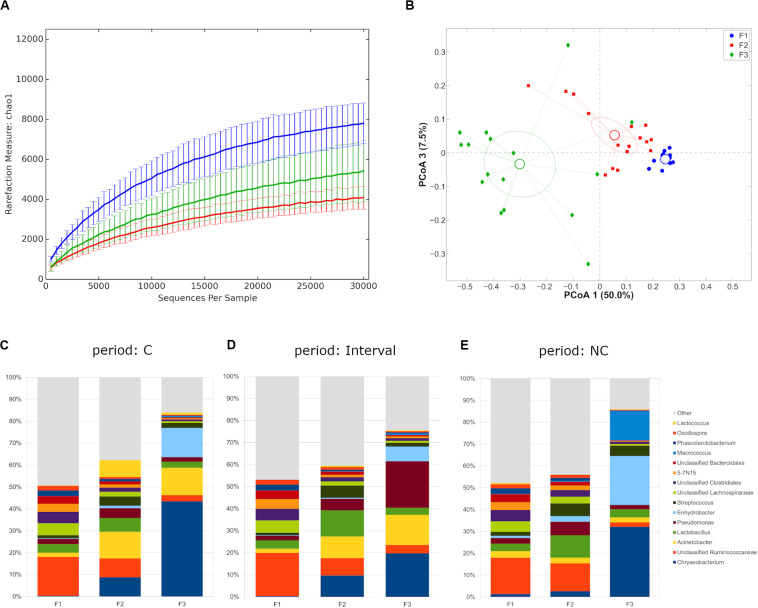
**(A)** Alpha-diversity rarefaction curves (Chao1 metric) for milk samples from different farms. Data points were averaged on all time points for each farm. For each curve, average and standard deviation are shown; **(B)** Principal Coordinates Analysis from weighted UniFrac distances among samples. Each point represents a sample; data points were colored according to the farm they were collected from. Centroids represent the average coordinate for the data points in each category and ellipses indicate the 95% confidence intervals. The first and third principal coordinates are represented; **(C–E)** Barplots of average microbial relative abundance at genus level for the three farms tested divided on the three experimental periods [i.e., chlorine (C), interval, non-chlorine (NC)].

Rarefaction curves evaluation suggested that the depth of coverage was enough to describe the biological diversity within the samples. Farms showed a different microbial diversity, with F1 showing the highest and F2 the lowest (*p*-value = 0.003 with chao1, Shannon and observed species metrics, Figure 1A). Major differences in the principal constituents of the microbial community were revealed (*p* = 0.001 in all pair-wise comparisons for both unweighted and weighted UniFrac distances) (Figure 1B), even separating along the time points (C, interval, NC), with the only exception of F2-F3 profiles during interval period (Supplementary Figure S1). Independently from the experimental period a distinctive microbiota composition for the three farms was highlighted, with F1 microbiota mainly constituted by Firmicutes (average relative abundance: 60.2%), whereas F2 showed a somehow higher abundance of Proteobacteria (24.5%), and F3 was dominated by Bacteroidetes (37.2%) and Proteobacteria (35.7%) (Supplementary Figure S2A). This difference was evident also at genus level, with F1 mainly characterized by unclassified members of *Ruminococcaceae* and *Lachnospiraceae* families, F2 by unclassified members of *Ruminococcaceae*, *Lactobacillus* spp., and *Acinetobacter* spp., and F3 by *Chryseobacterium* spp., *Enhydrobacter* spp. and *Acinetobacter* spp. (summing up to about 70% of average rel. ab.) (Figures 1C–E and Supplementary Figure S2B).

**FIGURE 2:**
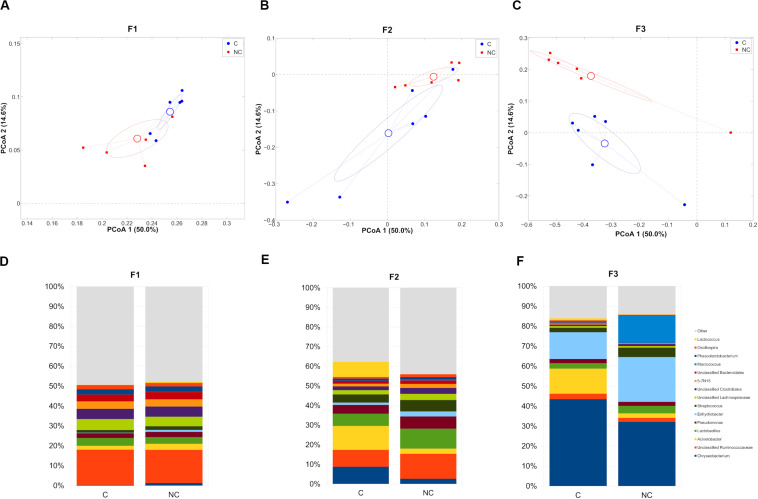
**(A–C)** Principal Coordinates Analysis from weighted UniFrac distances among samples divided on the farm they were collected from. Each point represents a sample; data points were colored according to the experimental period (only C and NC periods). Centroids represent the average coordinate for the data points in each category and ellipses indicate the 95% confidence intervals. The first and third principal coordinates are represented. **(D–F)** Barplots of average microbial relative abundances at genus level for chlorine and non-chlorine experimental periods divided on the three farms tested.

We, then, decided to exclude the “interval” period for further evaluations, since the microbiota was still in a transitional stage and we preferred focusing on the main differences after any possible microbiota evolution in the shift from C to NC detergent. The microbial composition in the two experimental periods (C and NC) for each farm was significantly different (*p* < 0.05, weighted UniFrac for all farms) (Figures 2A–C). In the shift from C to NC period, F1 microbial composition showed a significant increase of *Chryseobacterium* and a significant reduction of *Oscillospira*; F2, on the other hand, was characterized by a significant increase in *Oscillospira* and *Clostridium*, and by a tendency toward an increase of *Lactobacillus* and toward a reduction of *Lactococcus*; finally, F3 showed a significant reduction of *Acinetobacter* and a trend toward the increase of *Streptococcus* (Figures 2D–F).

**FIGURE 3:**
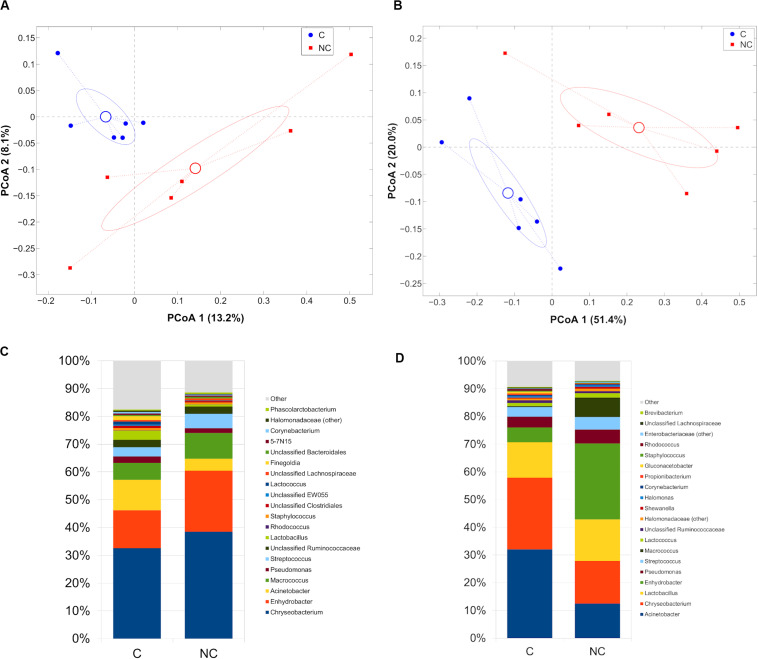
Principal Coordinates Analysis from **(A)** unweighted UniFrac distances among bulk milk samples and **(B)** weighted UniFrac distances among vat milk samples. Each point represents a sample; data points were colored according to the experimental period (only C and NC periods). Centroids represent the average coordinate for the data points in each category and ellipses indicate the 95% confidence intervals. Barplots of average microbial relative abundances at genus level for chlorine and non-chlorine experimental periods for **(C)** bulk milk and **(D)** vat milk samples.

Difference between C and NC periods was evidenced also in bulk and vat milk microbiota during Trentingrana cheese-making procedure. Analysis of intra- vs. inter- period sample distances (i.e., distances among samples from the same experimental period vs. distances from those of the other period) revealed a significant separation for bulk (*p* = 0.002, unweighted UniFrac) and vat milk (*p* = 0.001 for both weighted and unweighted UniFrac) (Figures 3A,B). In NC period, bulk and vat milk samples both showed an increase of *Enhydrobacter* and a reduction of *Acinetobacter*; bulk milk also showed an increase of *Chryseobacterium* and a reduction of *Lactobacillus*, whereas vat milk showed a decrease of *Chryseobacterium* and *Macrococcus* (Figures 3C,D). Microbial composition of bulk and vat milk resulted significantly different (*p* = 0.001 and *p* = 0.025 for unweighted and weighted UniFrac, respectively, Supplementary Figure S3A); the natural creaming favored the development of a more diverse microbial profile, as evident from that distances among bulk milk samples were significantly lower (*p* = 0.007, unweighted UniFrac) than those among vat samples (Supplementary Figure S3B) and by that bulk-vat paired distances did not result different from those between unpaired samples (i.e., distance between each bulk sample and all vat except from its paired one) (Supplementary Figure S3C,D).

**FIGURE 4:**
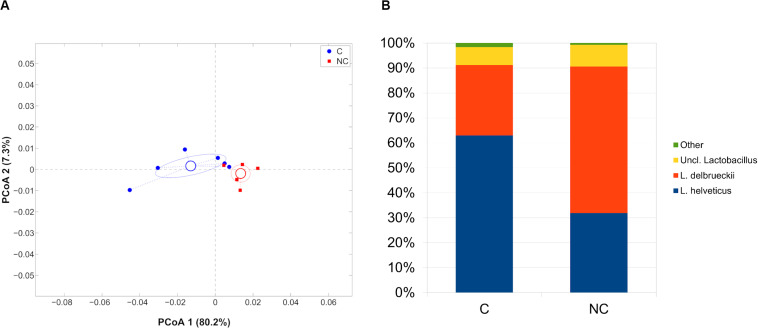
**(A)** Principal Coordinates Analysis from weighted UniFrac distances among whey starter samples. Each point represents a sample; data points were colored according to the experimental period (only C and NC periods). Centroids represent the average coordinate for the data points in each category and ellipses indicate the 95% confidence intervals. The first and second components of the variance are shown. **(B)** Barplots of average microbial relative abundances of *Lactobacillus* species for chlorine and non-chlorine experimental periods.

Finally, whey-starter composition was quite entirely composed by *Lactobacillus* (98.8% of relative abundance), as expected; however, microbial profiles in C and NC periods remained distinct (*p* = 0.006, weighted UniFrac). At species-level, *Lactobacillus helveticus* was predominant during period C (62.9% of relative abundance) and significantly reduced during period NC (31.8%, *p* = 0.008), whereas *L. delbrueckii* had an opposite trend (28.1 and 58.6% for C and NC periods, respectively; *p* = 0.004) (Figure 4).

### Cheese Results

**FIGURE 5:**
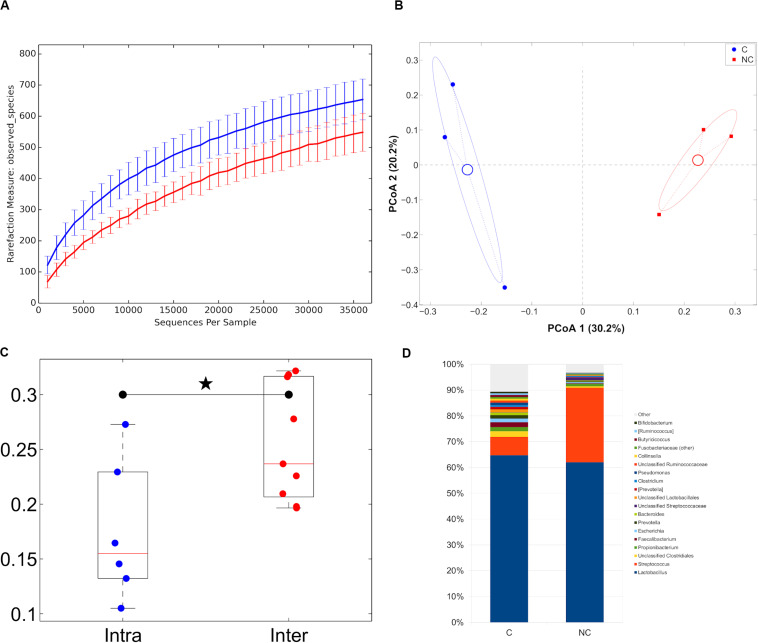
**(A)** Alpha-diversity rarefaction curves (observed_species metric) for cheese samples representative of the experimental periods (C and NC). For each curve, average and standard deviation are shown; **(B)** Principal Coordinates Analysis from unweighted UniFrac distances among cheese samples. Each point represents a sample; data points were colored according to the experimental period. Centroids represent the average coordinate for the data points in each category and ellipses indicate the 95% confidence intervals. The first and second principal coordinates are represented; **(C)** Boxplots of intra- and inter-sample distances for chlorine and non-chlorine experimental periods; **(D)** Barplots of average microbial relative abundances at genus level for chlorine and non-chlorine experimental periods for cheese samples.

With regard to cheese analysis, the sequencing was characterized by a total of 379,433 high quality reads, with a mean of 63,239 ± 14,054. Despite a small number of samples analyzed, both alpha- and beta-diversity analysis revealed a trend toward a separation between the two treatments (C and NC cheese samples), as it was evidenced by the significant difference in weighted UniFrac distances of cheese samples characterized by the same detergent vs. those of the other detergent (Figures 5A–C).

Trentingrana DOP cheese microbiota for NC treatment samples was dominated by members of the Firmicutes phylum, which accounted for about 96% of the relative abundance, while samples with C treatment revealed a composition made by a lower presence of Firmicutes (86.3% on average) and by Actinobacteria (3.7%), Proteobacteria (4.0%) and Bacteroidetes (4.4%). The overall bacterial composition of Trentingrana DOP cheese showed two main genera: *Lactobacillus* spp. (64.7 and 62.0% for C and NC, respectively) and *Streptococcus* spp. (7.1 and 28.9% for C and NC, respectively), together representing >90% of the total abundance in NC samples. Moreover, NC samples had lower presence of *Faecalibacterium* spp., *Propionibacterium* spp., and *Escherichia* spp. (Figure 5D).

Since *Streptococcus* and *Lactobacillus* genera constituted more than 90 and 70% of the total bacterial population for NC and C samples, respectively, a focus on these genera has been performed. Among *Lactobacillus*, *L. helveticus*, and *L. delbrueckii* were the main species in NC samples while *L. helveticus* and unclassified *Lactobacillus* were the predominant in C samples. *Streptococcus* population was dominated by *S. thermophilus* in both the experimental conditions (C and NC samples) (Supplementary Figure S4).

### Volatilome

Supplementary Table S1 reports the relative abundance of the 19 main volatile metabolites detected in Trentingrana cheese samples, including six acids (acetic, propanoic, butanoic, 3-methylbutanoic, pentanoic, and hexanoic acid), three alcohols (ethanol, butan-1-ol, and pentan-2-ol), two aldehydes (2-methylbutanal and 3-methylbutanal), three esters (ethyl acetate, ethyl butyrate, and ethyl hexanoate), and five ketones (acetone, butan-2-one, pentan-2-one, heptan-2-one, and 3-hydroxybutan-2-one).

As expected, there was a strong difference between peripheral and inner portions of the cheeses, due to the heat load in the cheese core during molding that affects the ripening process (Pellegrino et al., 1997a,b). As a result, most of the carbonyl compounds and alcohols were more abundant in the inner samplings (*p* range: 0.002–0.037), while acids deriving from lipolysis and esters were more abundant in the peripheral ones (*p* range: <0.001–0.003).

Regarding C and NC periods, volatilome analysis showed an almost similar profile, with exception of butan-1-ol, propanoic and hexanoic acid that resulted to be significantly influenced by the two treatments applied (Table 3). Cheeses obtained in the C period were characterized by a higher level of propanoic acid, both in the inner and peripheral portions (*p* = 0.002 and 0.050, respectively). Moreover, only in the inner part, during C period butan-1-ol level was higher (*p* = 0.06) and hexanoic acid level was lower (*p* = 0.001) with respect to the corresponding NC period.

**TABLE 3:** Volatile organic compound (VOC) significantly affected by chlorine and non-chlorine treatment in Trentingrana cheese samples after 12 months of ripening at.

**Compounds**	Odor description*	**Sampling**	**Chlorine**	**No-chlorine**	**Pooled SD**	***p*-value**
Butan-1-ol	fruity	inner	5.13^B^	4.49^A^	0.32	0.006
		peripheral	4.00	3.87	1.18	0.856
Propanoic acid	sour	inner	5.07^B^	4.28^A^	0.32	0.002
		peripheral	4.94^B^	4.35^A^	0.45	0.050
Hexanoic acid	rancid	inner	6.21^A^	6.44^B^	0.09	0.001
		peripheral	6.85	6.89	0.08	0.384

*Odor description reported derived from Curioni and Bosset (2002). Statistically significant differences in rows (*p* ≤ 0.05) are indicated by different superscripts. Data expressed as log10 of arbitrary units (AU) of the peak area of the characteristic ion.

## Discussion

The purpose of this study was to investigate the influence of the use of chlorine-based products in the milking cleaning routine at farm level on bulk tank milk microbiota, and their impact on the dairy processing capacity. Furthermore, information supporting the importance of the farm environment as a unique environment was also provided.

In our study, the three farms were located in a narrow area (about 10 km from one another), had similar management system (herd entity and composition, herd housing and health, hygiene, udder preparation, milking equipment, cleaning and disinfecting procedures) but, despite this, they were characterized by a distinctive microbiota.

In fact, while cultural analysis revealed only slightly not significant differences between the microbial counts of bulk milks, alpha- and beta-diversities of milk microbiota samples provided a clear separation between samples from the three farms, demonstrating that the communities of milk microbiota were highly diverse. This observation clearly supports what different authors affirmed in previous studies, that multiple key factors characterizing the single farm and the exposition to niche-specific microbes during winter indoor housing, significantly influence the microbial community composition of bulk milk (Verdier-Metz et al., 2009; Mallet et al., 2012; Doyle et al., 2017; Li et al., 2018). Consistent with previous studies, the major bacterial phyla detected in the milk samples were Actinobacteria, Bacteroidetes, Firmicutes and Proteobacteria (Joishy et al., 2019); nevertheless, the relative abundance of useful cheese-making and spoilage micro-organisms differed from one farm to the other, with important implications from a dairy perspective. In F1 and F2 microbial taxonomic composition was dominated by Firmicutes (rel. ab. respectively 60.2% and 47.3%), while farm F3 showed a microbial composition dominated by Bacteriodetes (37.2%) and Proteobacteria (35.7%). NGS analysis revealed that farms F1 and F2 were richer in LAB (*Lactobacillus* and *Lactococcus* genera) while *Chryseobacterium*, and *Acinetobacter*, dairy spoilage associated genera (Vithanage et al., 2016), dominated in F3 milk samples.

The non-chlorine cleaning did not cause any increase in coliforms and staphylococci count, confirming that hot water usage, along with the proper use of non-chlorine products, assure the hygienic quality of milk (Gleeson et al., 2013). Our results are consistent with those reported by Pandey et al. (2014), which did not find significant differences in microbial counts comparing 200 ppm chlorine solution and 50 ppm iodophore solution usage for cleaning milking equipment.

Chlorine is known to alter both Gram-negative and Gram-positive bacterial membrane permeability providing cellular degeneration (Muhandiramlage et al., 2020). It is reasonable to hypothesize that its use in milking equipment cleaning and sanitation can influence the biodiversity of raw milk-associated microbiota.

Although the three bulk milk samples still contained a reproducible distinctive microbiota, bacterial population (beta-diversity) was different within each farm according to the chlorine use in cleaning milking equipment. Metagenomic analysis highlighted that the influence of chlorine use on microbial population composition is still detectable for several days after the interruption of its use. Comparing C and NC periods, relative abundances analysis revealed that farm F2 and F3 had a significant decrease in *Chryseobacterium* and *Acinetobacter* genera, while in F1 milk the main reduction was observed regarding *Oscillospira* genus. Differently, *Chryseobacterium* and *Acinetobacter* increased in F1 milk moving from C to NC period, whereas in F2 the largest increase was in *Oscillospira*, *Clostridium*, *Lactobacillus*, and *Ruminococcaceae* families and in F3 a noticeable increase with regard to *Streptococcus*, *Macrococcus*, and *Enhydrobacter* was observed.

The different impact of chlorine products usage on the composition of raw milk microbial population is of great interest for dairy related processes. *Chryseobacterium* and *Acinetobacter* are Gram-negative bacteria associated to dairy spoilage, while *Oscillospira* is a gut-related genus belonging to *Ruminococcaceae* family (Joishy et al., 2019). *Chryseobacterium*, *Acinetobacter*, and *Clostridium* can cause a negative effect on the product quality, differently by *Lactobacillus*, *Lactococcus*, and *Streptococcus* which are the main actors of the cheesemaking process of raw milk cheeses. *Macrococcus* and *Enhydrobacter* have been previously reported as core bacterial genera in naturally fermented dairy products, although their role in the definition of quality attributes is still not clear (Mallet et al., 2012; Quigley et al., 2013; Zhong et al., 2016; Joishy et al., 2019).

Non-chlorine cleaning protocols have been set up and validated to reduce chlorine residues in milk and in derived dairy products (Gleeson et al., 2013). Different studies demonstrated that chlorine is highly effective in reducing the bacterial population in milk (Pandey et al., 2014), but no information is available on its influence on the diversity and microbiota of raw milk.

Bacterial populations in raw milk consisting of the union of the three bulk milks arriving at dairy processing plant was analyzed before and after natural creaming. Natural creaming of milk in a large flat vat for about 12 h occurring at environmental temperature is known to largely affect the bacterial population of milk (Franciosi et al., 2012). As expected, high-throughput DNA sequencing analysis evidenced a significant microbial diversity in whole milk compared to partially skimmed milk. In addition, the impact of chlorine products use at farm level on milk microbiome composition after maturation was highlighted by the metagenomic data: significant changes in the core microbiota were observed with a reduction of *Acinetobacter*, *Chryseobacterium*, and *Macrococcus* genera and an increase with regard to *Enhydrobacter*.

As expected, whey-starter bacterial composition consisted mainly of *Lactobacillus* genus (Rossetti et al., 2008; Gatti et al., 2014; Morandi et al., 2019; Bertani et al., 2020). At a species-level, *L. helveticus* was predominant during period C (62.9% rel. ab.) and significantly reduced during period NC (31.8%), whereas *L. delbrueckii* had the exact opposite trend (28.1% period C; 58.6% period NC). This result is noteworthy, since we have already reported a worrying reduction of presence of several *Lactobacillus* species, foremost *L. delbrueckii*, in whey starter for Trentigrana production (Morandi et al., 2019). The loss of microbial biodiversity has been associated to a depletion of raw milk cheese sensory attributes by many authors (Broadbent et al., 2011; De Filippis et al., 2014).

Microbial diversity in cheese produced with milk collected from the three farms during C and NC experimental periods was found to be diverse, indicating the impact on the indigenous milk bacteria and in shaping cheese bacterial composition and consequently cheese quality traits.

Alpha- and beta-diversity analyses revealed significant differences among C and NC cheeses. The main differences found in the abundance of microbial groups concerned the Firmicutes taxa, which increased from 86.3 to 96% moving from C to NC period. The observed increase in the Firmicutes content is due to an increment in LAB abundances, in particular *Lactobacillus* and *Streptococcus* genera, that represent the key actors of the Trentingrana cheese making process for their acidifying activity in milk and their proteolytic activity in cheese, along with aroma compounds production (Morandi et al., 2019). Among *Lactobacillus* genus, as a consequence of previously underlined diversified whey starter microbial composition, a higher presence of *L. delbrueckii* was observed when chlorine products were not used at farm level. This result is of particular importance since LAB biodiversity has been associated to a higher sensory quality in cheese by different studies (Pogačić et al., 2016; Morandi et al., 2019).

This is consistent with data on the volatilome analysis of Trentingrana experimental wheels as it showed a slightly less intense lipolysis during C period in the inner part of the wheel cheese. In particular, hexanoic acid, one of the most important flavor compounds of Grana Padano cheese (Curioni and Bosset, 2002), showed significantly higher levels in NC samples. This compound derives from the lipolysis of milk triglycerides and, depending on its concentration and perception threshold, can contribute positively or negatively to the cheese aroma, being part of its typicity or resulting as a rancidity defect (Collins et al., 2003). Propanoic acid, responsible for sour notes, typically originated from the fermentation of the lactic acid produced by LAB during the cheese-making, showed a significantly higher level in the C period than in the NC period, confirming a higher development of Actinobacteria, in particular of *Propionibacterium*. Finally, in C samples a high level of butan-1-ol was detected. This compound has been identified in Grana Padano cheese and has been described as having floral, fragrant, fruity, sweet notes (Gómez-Torres et al., 2015) but, due to its high sensory threshold, slightly contributes to cheese aroma (Qian and Burbank, 2007).

## Conclusion

Raw milk microbial population is critically related to milk processability, spoilage and safety characteristics, but also plays a primary role in the deriving raw milk cheese quality, safety and sensory attributes.

To better understand the major drivers affecting the composition of milk microbiota at farm level, different studies have been conducted through both culture-based and high-throughput DNA sequencing technologies. In our study, we provided some evidences deepening the relationship between the use of chlorine products in cleaning and sanitizing procedure at farm level, microbial community of raw milk and its impact on whey starter and cheese microbiome.

Our preliminary results indicate that chlorine replacement is not associated with an increase of spoilage bacteria, staphylococci and coliforms, but it leads to an increase the milk microbiota biodiversity and, consequently, it can improve raw milk production performances and the overall cheese quality.

Further studies on cleaning and sanitation strategies alternative to chlorine-based protocols are needed, in order to define both their periodicity and regularity of application.

## Data Availability Statement

The datasets presented in this study can be found in online repositories. The names of the repository/repositories and accession number(s) can be found in the article/ Supplementary Material.

## Author Contributions

MB, AG, MM, and LZ conceived and planned the experiments. AG collected the milk, whey and cheese samples. PC, NC, and GB carried out the experiments. BC, MB, MM, EP, and LZ contributed to the experiments and the interpretation of the results. CC, MS, and AT performed the statistical analyses. PC, SM, MS, and MB wrote the manuscript in consultation with BC and GB. All authors discussed the results and critically revised and approved the final manuscript.

## Conflict of Interest

AG and NC were employed by the Trentingrana–Consorzio dei Caseifici Sociali Trentini s.c.a., Trento, Italy.

The remaining authors declare that the research was conducted in the absence of any commercial or financial relationships that could be construed as a potential conflict of interest.

## Footnotes

1 ftp://greengenes.microbio.me/greengenes_release/gg_13_5/gg_13_8_otus.tar.gz

2 ftp://ftp.ncbi.nlm.nih.gov/genomes/refseq/bacteria/

3 http://www.ncbi.nlm.nih.gov/sra

## Supplementary Material

Supplementary Figure 1 Principal Coordinates Analysis from weighted UniFrac distances among samples divided on the experimental period (**A:** chlorine, **B:** interval, **C:** non-chlorine). Each point represents a sample; data points were colored according to the farm they were collected from. Centroids represent the average coordinate for the data points in each category and ellipses indicate the 95% confidence intervals. The first and third principal coordinates are represented.

Supplementary Figure 2 Barplots of bacterial relative abundances at **(A)** phylum and **(B)** genus level. Each bar represents a sample; samples are sorted according to farm and experimental period. Colored lines below each plot indicate the experimental period (blue: chlorine; green: interval; red: non-chlorine). Only the nine most abundant phyla and the 24 most abundant genera were shown. All remaining taxa were summed up into the “Other” category.

Supplementary Figure 3 **(A)** Principal Coordinates Analysis from unweighted UniFrac distances among bulk and vat milk samples. Each point represents a sample; data points were colored according to their source, independently from the experimental period. Centroids represent the average coordinate for the data points in each category and ellipses indicate the 95% confidence intervals; **(B)** Boxplots of paired unweighted UniFrac distances. For each experimental period, distances between paired bulk and vat milk samples are represented. The black star indicates a statistically significant difference (Mann–Whitney *U*-test, *p* < 0.05); **(C,D)** Boxplots of paired bulk-vat milk intra- and inter-sample distances for chlorine and non-chlorine experimental periods.

Supplementary Figure 4 Barplots of bacterial relative abundances for *Lactobacillus* and *Streptococcus* species-level characterization. All other bacteria are grouped into “Other” category. **(A)** Barplots of single samples (chlorine: left-most samples; non-chlorine: right-most samples) and **(B)** average relative abundances for experimental period.
