# Supplementary material for: Raw Milk Microbiota Modifications as Affected by Chlorine Usage for Cleaning Procedures: The Trentingrana PDO Case
Source: Front Microbiol. 2020 Oct 6;11:564749. doi: 10.3389/fmicb.2020.564749 (PMC7573252; doi:10.3389/fmicb.2020.564749)
Supplement: Supplementary Figure 1 — Principal Coordinates Analysis from weighted UniFrac distances among samples divided on the experimental period (A: chlorine, B: interval, C: non-chlorine). Each point represents a sample; data points were colored according to the farm they were collected from. Centroids represent the average coordinate for the data points in each category and ellipses indicate the 95% confidence intervals. The first and third principal coordinates are represented. [file Data_Sheet_1.pdf]

Supplementary Material

1.1 Supplementary Figures

Supplementary Figure 1 Principal Coordinates Analysis from weighted Unifrac distances among samples divided on the experimental period (A: chlorine, B: interval, C: non-chlorine). Each point represents a sample; data points were colored according to the farm they were collected from. Centroids represent the average coordinate for the data points in each category and ellipses indicate the 95% confidence intervals. The first and third principal coordinates are represented.

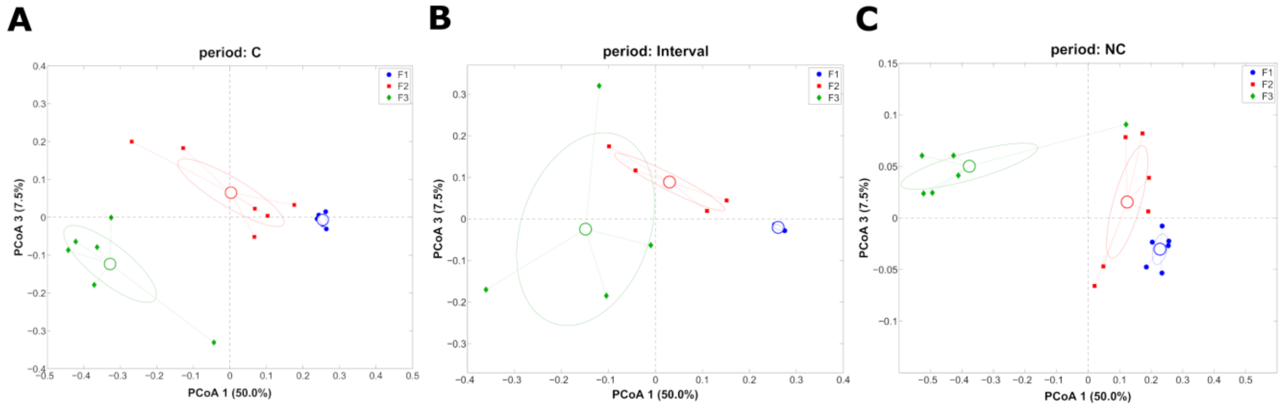

Supplementary Figure 2 Barplots of bacterial relative abundances at (A) phylum and (B) genus level. Each bar represents a sample; samples are sorted according to farm and experimental period. Colored lines below each plot indicate the experimental period (blue: chlorine; green: interval; red: non-chlorine). Only the 9 most abundant phyla and the 24 most abundant genera were shown. All remaining taxa were summed up into the "Other" category.

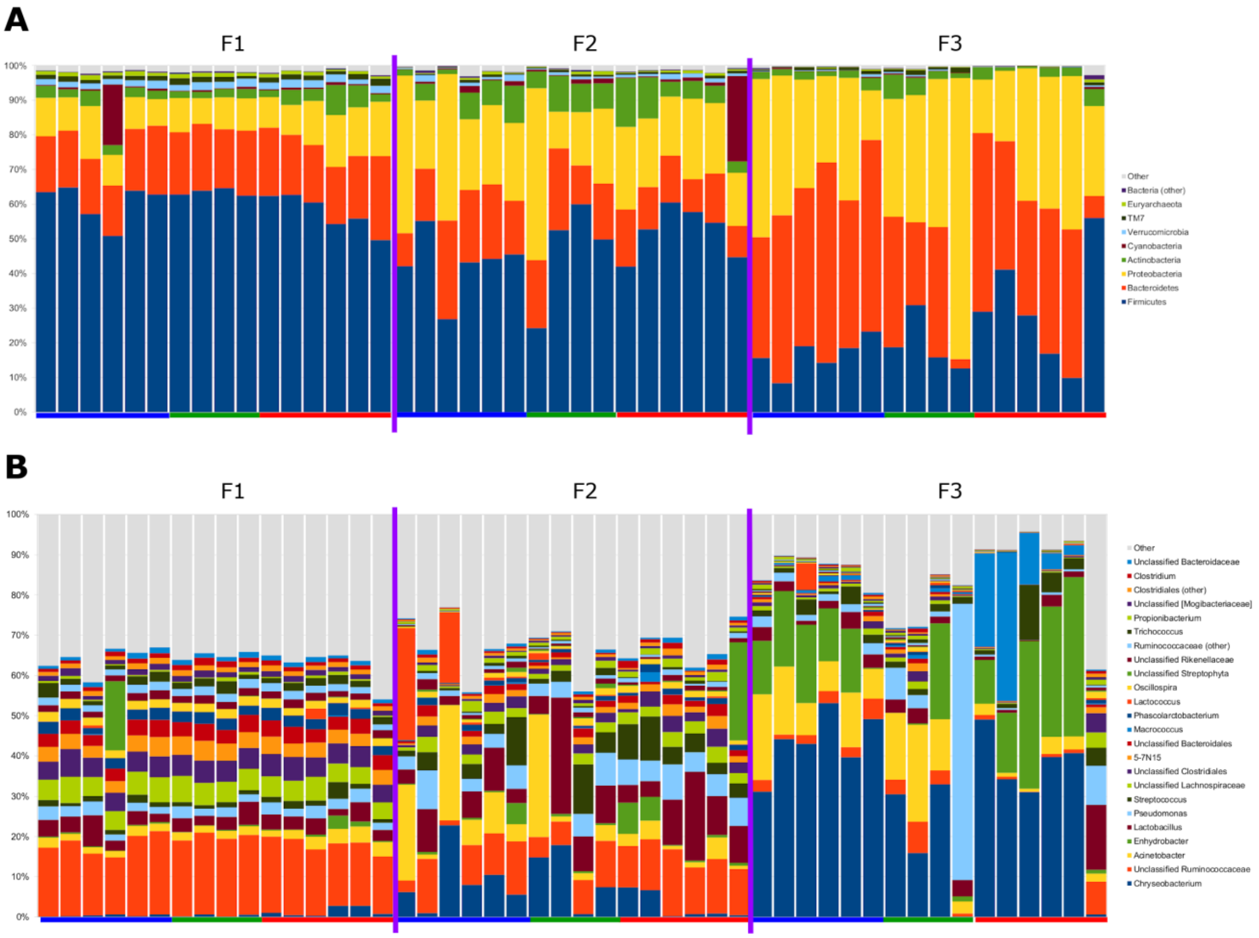

Supplementary Figure 3 (A) Principal Coordinates Analysis from unweighted Unifrac distances among bulk and vat milk samples. Each point represents a sample; data points were colored according to their source, independently from the experimental period. Centroids represent the average coordinate for the data points in each category and ellipses indicate the 95% confidence intervals; (B) Boxplots of paired unweighted Unifrac distances. For each experimental period, distances between paired bulk and vat milk samples are represented. The black star indicates a statistically significant difference (Mann-Whithney U-test, p<0.05); (C-D) Boxplots of paired bulk-vat milk intra- and inter- sample distances for chlorine and non-chlorine experimental periods.

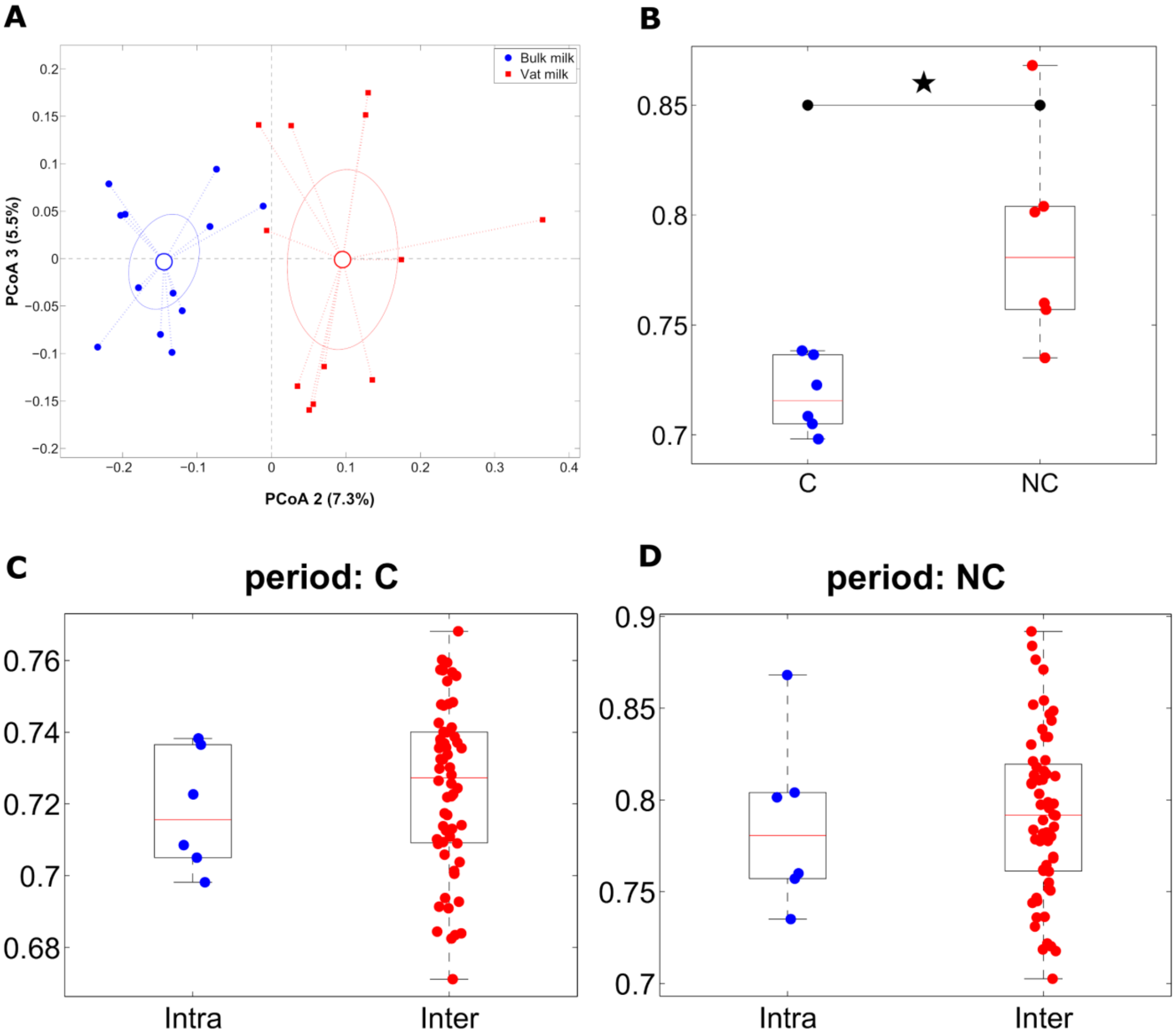

Supplementary Figure 4 Barplots of bacterial relative abundances for *Lactobacillus* and *Streptococcus* species-level characterization. All other bacteria are grouped into "Other" category. (A) Barplots of single samples (chlorine: left-most samples; non-chlorine: right-most samples) and (B) average relative abundances for experimental period.

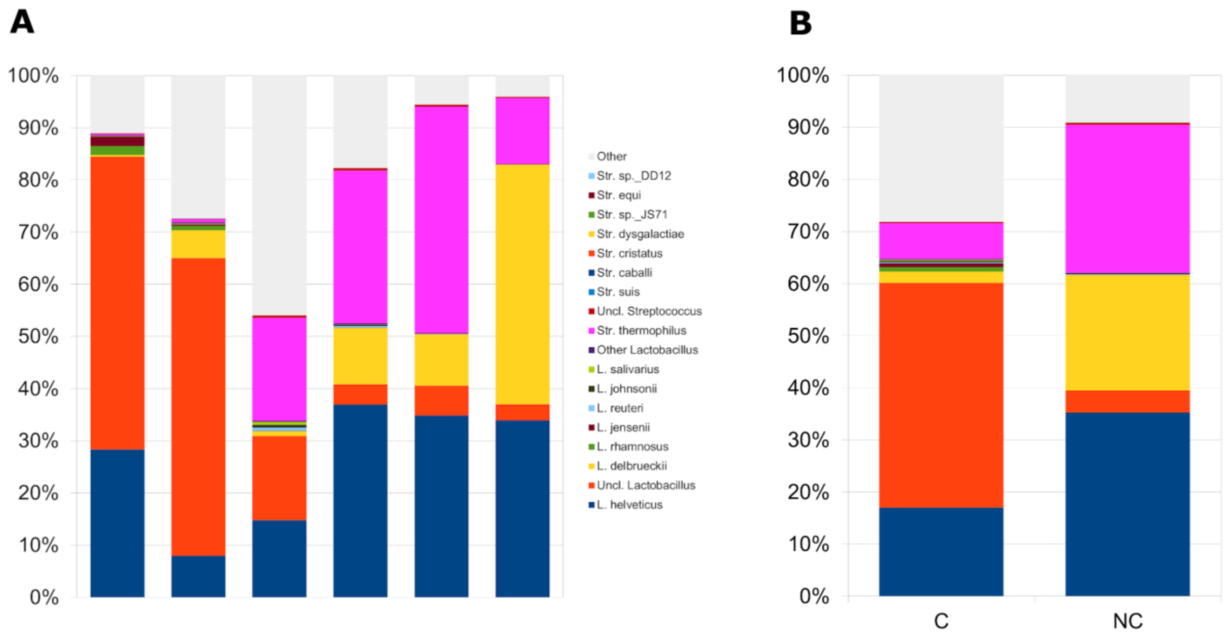

1.2 Supplementary Tables

Supplementary Table 1 - Volatile organic compounds detected in Trentingrana cheese samples at 12 months of ripening. Data expressed as $\log_{10}$ of arbitrary units (AU) of the peak area of the characteristic ion. Each value is the mean of 2 determinations

**Classes**	**Compounds**	**Sampling**	**Chlorine**	**No-chlorine**	**Pooled SD**	***p* value**
Acids	Acetic acid	inner	6,54	6,59	0,12	0,458
		peripheral	6,59	6,58	0,10	0,814
	Propanoic acid	inner	$5,07^{B}$	$4,28^{A}$	0,32	0,002
		peripheral	$4,94^{B}$	$4,35^{A}$	0,45	0,050
	Butanoic acid	inner	6,81	6,81	0,07	0,918
		peripheral	7,15	7,15	0,05	0,966
	3-methylbutanoic acid	inner	4,83	4,81	0,44	0,936
		peripheral	4,87	4,71	0,20	0,201
	Pentanoic acid	inner	4,49	4,52	0,10	0,536
		peripheral	4,91	4,90	0,05	0,952
	Hexanoic acid	inner	$6,21^{A}$	$6,44^{B}$	0,09	0,001
		peripheral	6,85	6,89	0,08	0,384
Alcohols	Ethanol	inner	6,12	6,09	0,20	0,765
		peripheral	6,21	6,05	0,20	0,192
	Butan-1-ol	inner	$5,13^{B}$	$4,49^{A}$	0,32	0,006
		peripheral	4,00	3,87	1,18	0,856
	Pentan-2-ol	inner	5,55	5,71	0,25	0,284

		peripheral	4,95	5,43	0,53	0,146
Aldehydes	2-Methylbutanal	inner	4,86	4,94	0,10	0,212
		peripheral	4,90	4,86	0,12	0,494
	3-Methylbutanal	inner	5,12	5,12	0,10	0,961
		peripheral	5,03	4,96	0,12	0,300
Esters	Ethyl acetate	inner	5,16	5,19	0,24	0,803
		peripheral	5,18	5,08	0,25	0,514
	Ethyl butyrate	inner	5,32	5,22	0,20	0,382
		peripheral	5,63	5,47	0,20	0,195
	Ethyl hexanoate	inner	4,62	4,77	0,30	0,403
		peripheral	5,43	5,32	0,24	0,443
Ketones	Acetone	inner	6,04	5,92	0,19	0,280
		peripheral	5,91	5,71	0,30	0,267
	Butan-2-one	inner	5,34	5,20	0,14	0,098
		peripheral	5,20	5,01	0,19	0,116
	Pentan-2-one	inner	6,28	6,21	0,15	0,480
		peripheral	6,01	5,84	0,35	0,439
	Heptan-2-one	inner	5,92	5,90	0,07	0,678
		peripheral	5,85	5,62	0,25	0,164
	3-Hydroxybutan-2-one	inner	5,65	5,54	0,21	0,415
		peripheral	5,17	5,11	0,43	0,792
